# Overexpression of MyrAkt1 in Endothelial Cells Leads to Erythropoietin- and BMP4-Independent Splenic Erythropoiesis in Mice

**DOI:** 10.1371/journal.pone.0055095

**Published:** 2013-01-28

**Authors:** Rebekah K. O’Donnell, Whitney E. Goldstein, Carole Perruzzi, Laura E. Benjamin, William Aird

**Affiliations:** 1 Department of Pathology, Beth Israel Deaconess Medical Center, Boston, Massachusetts, United States of America; 2 Center for Vascular Biology Research, Beth Israel Deaconess Medical Center, Boston, Massachusetts, United States of America; 3 Division of Molecular and Vascular Medicine, Department of Medicine, Beth Israel Deaconess Medical Center, Boston, Massachusetts, United States of America; University Magna Graecia, Italy

## Abstract

Under steady state conditions, erythropoiesis occurs in the bone marrow. However, in mice, stress or tissue hypoxia results in increased erythropoiesis in the spleen. There is increasing evidence that the hematopoietic microenvironment, including endothelial cells, plays an important role in regulating erythropoiesis. Here, we show that short-term expression of constitutively active Akt in the endothelium of mice results in non-anemic stress erythropoiesis in the spleen. The initiation of this stress response was independent of erythropoietin and BMP4, and was observed in endothelial myrAkt1 mice reconstituted with wild-type bone marrow. Together, these data suggest that endothelial cell hyperactivation is a potentially novel pathway of inducing red cell production under stress.

## Introduction

Erythropoiesis is a multistep process that involves the differentiation of early pluripotent hematopoietic stem cells through a series of lineage-committed cells including erythroid burst-forming unit (BFU-E) and erythroid colony-forming unit (CFU-E) progenitor cells. Under steady state conditions, erythropoiesis occurs in the bone marrow. However, in mice, stress or tissue hypoxia results in increased erythropoiesis in the spleen. This response, termed *stress erythropoiesis*, involves the rapid proliferative response of a population of erythropoietic progenitor cells. Recent studies on stress erythropoiesis have defined it as a qualitatively different process from steady-state erythropoiesis, and have identified a subset of progenitors that are specific to the stress response [1], [2]. The differentiation of progenitors in both pathways is dependent upon erythropoietin, but stress erythropoietic progenitors appear to also require bone morphogenetic protein 4 (BMP4) for expansion in the spleen [3]–[6].

Akt1/Protein Kinase B (PKB) is a serine/threonine kinase that functions as a central node in the cellular signaling response to growth factors and other stimuli and plays an important role in a diverse array of downstream functions (reviewed in [7]). Akt1 is activated by phosphorylation at the plasma membrane. Constitutive overexpression of Akt1 can be achieved by signal-independent membrane targeting with a myristoylation sequence (myrAkt1). A previous study described the generation of a double transgenic mouse model that expresses myrAkt1 in endothelial cells under tetracycline control [8]. Two independent lines of mice were generated, one with low levels of expression, the other with high levels of expression. Sustained expression of dominant active myrAkt1 in the intact endothelium of mice resulted in enlarged, hyperpermeable blood vessels that mimic those of tumors [9], [10]. The latter phenotype was observed at 6–7 weeks following the withdrawal of tetracycline in the lower expressing line.

Recently, it was reported that Akt activation in endothelial cells resulted in an increased number of total hematopoietic cells in the bone marrow and spleen [11]. Mice transplanted with bone marrow cells from the myrAkt1 mice displayed rapid hematopoietic recovery. Moreover, bone marrow cells from the overexpressing mice had a competitive advantage in long-term, multi-lineage engraftment, compared with cells from wild-type mice. Thus, endothelial cell-specific Akt1 activation in endothelial cells plays a role in the reconstitution of hematopoietic stem and progenitor cells.

In the current study, we were interested in determining the effect of short-term expression of constitutively active Akt1 in the endothelium. We found that endothelial myrAkt1 mice developed non-anemic stress erythropoiesis in the spleen. The initiation of this stress response was independent of erythropoietin and BMP4, and was observed in endothelial myrAkt1 mice reconstituted with wild-type bone marrow. Together, these data suggest that endothelial cell hyperactivation is a potentially novel pathway of inducing red cell production under stress.

## Materials and Methods

### Ethics Statement

All animal studies were carried out in accordance with the recommendations in the Guide for Care and Use of Laboratory Animals of the National Institutes of Health. The protocol was approved by the Beth Israel Deaconess Medical Center Institutional Animal Care and Use Committee (protocol 016-2010). All surgery was performed under anesthesia, and all efforts were made to minimize suffering.

### Experimental Mice

The double transgenic mouse model that expresses myrAkt1 in endothelial cells under tetracycline control has been previously described [8]. Briefly, the mice carry a transgenic construct (VEcadherin:tTA) in which the endothelial–specific VE-cadherin promoter is coupled to the tetracycline-regulated transcriptional activator (tTA) gene and another construct (TET:myrAkt1) in which myrAkt (full-length Akt1 with a c-Src myristoylation sequence and hemagglutinin tag added to the N terminus) is under the control of a tetracycline (TET)-responsive promoter. To suppress myrAkt1 expression in embryos and newborn mice, pregnant and lactating females were provided with 1.5 mg/mL tetracycline/5% sucrose in their drinking water. Mice continued to receive tetracycline until they reached a minimum of 8 weeks of age, at which time they were switched to regular water to induce myrAkt1 expression in the endothelium. Two lines of VEcadherin:tTA mice were used: the D5 line with higher expression and the D4 line with lower expression of tTA. Double transgenic D5 animals develop a vascular phenotype in 8–12 days after tetracycline withdrawal, while D4 animals require 6–8 weeks to develop a similar phenotype [8], [10]. BMP4+ macrophage recruitment was shown in an animal recovering from an i.p. injection of 5-fluorouracil, 250 mg/kg.

### Immunohistochemistry

Formalin-fixed, paraffin-embedded sections were retrieved with boiling citrate buffer. After endogenous peroxidase and nonspecific protein block (0.6% H_2_O_2_, 5% goat serum in PBS), slides were incubated with polyclonal TER119 antibody (1∶50 dilution; eBioscience, San Diego, CA) or monoclonal HP-1 antibody (1∶100 dilution; Hypoxyprobe, Inc., Burlington, MA) for one hour at 37°C. After incubation with secondary polyclonal antibody (goat anti-rat or anti-mouse, Vector Laboratories, Burlingame, CA) and streptavidin horseradish peroxidase (Vector Laboratories), staining was developed with 3-3-diaminobendizine (Life Technologies, Grand Island, NY). Slides were counterstained with hematoxylin. Peripheral blood smears were stained with the Hema3 Stat Pack (Fisher Scientific, Pittsburgh, PA). Frozen sections were stained with polyclonal BMP4 antibody (Abcam, Cambridge, MA) and Cy2-conjugated secondary polyclonal anti-rabbit antibody (Vector Laboratories), CD41-FITC antibody (eBioscience), and an In Situ Cell Death Detection kit (Roche Diagnostics, Indianapolis, IN). Images were acquired on a Zeiss Imager.A1 with a Zeiss AxioCam MRc using AxioVision software (Carl Zeiss MicroImaging, Thornwood, NY).

### Cell Preparations and Flow Cytometry Analysis

Mouse femurs were flushed and spleens were crushed in cold PBS or IMDM (Mediatech, Manassas, VA) and single-cell suspensions were prepared. Red cells were lysed with red cell lysis buffer (Quality Biological, Gaithersburg, MD) before FACS analysis. Cells were counted and incubated with Fc block (eBioscience), followed by antibodies against TER119, CD71, CD45, B220, IgM, IgD, CD24, CD43, CD4, CD8, CD11b, and Annexin V, conjugated with PE, FITC, APC, PE-Cy5, and PE-Cy7 (eBioscience). Cells were then acquired on a FACSCanto II (BD Biosciences) and analyzed using FACSDiva (BD Biosciences).

### Blood and Tissue Analysis

Blood was collected in heparinized tubes (BD Biosciences) and evaluated for hematocrit with a Hemavet (Drew Scientific, Waterbury, CT). Reticulocytes were evaluated using Retic-COUNT (BD Biosciences, San Jose, CA) according to the manufacturer’s instructions. Animals were temporarily anesthetized with 2% isofluorane from a precision vaporizer (Midmark, Versailles, OH) through a nose cone and oxygen saturation was measured in the thigh using a MouseOx oximeter (Starr Life Sciences, Oakmont, PA). Hypoxia was evaluated by i.p. injection of 60 mg/kg Hypoxyprobe-1 (Hypoxyprobe, Inc.) in 40–60 µl of PBS approximately one hour prior to sacrifice.

### Gene Expression and ELISA

RNA was isolated from tissues using Trizol (Invitrogen, Grand Island, NY) and cDNA synthesis was performed with the High Capacity cDNA Reverse Transcription Kit (Applied Biosystems, Grand Island, NY). RNA was quantified using Sybr Green (Applied Biosystems) on a 7500 Fast Real-Time PCR System (Applied Biosystems) using primers for erythropoietin (5′ TAGCTGCCGGAGCTCCTTA, 5′ CAGGCTAGTGGGGTGATCTG) and BMP4 (5′ TGCTTTTCGTTTCCTCTTCAACC, 5′ AAGTTTCCCACCGTGTCACA) from Integrated DNA Technologies (Coralville, IA). Values were normalized against 18S. Erythropoietin protein concentration in plasma was evaluated by ELISA (Quantikine Mouse Epo Kit, R&D Systems, Minneapolis, MN). Haptoglobin concentration (Immunology Consultants Laboratory, Portland, OR) and lactate dehydrogenase activity (Promega, Madison, WI) were evaluated in serum, according to the manufacturer’s instructions.

### Colony Assays

A total of 2^7^ nucleated spleen cells were plated in 1.1 mL of methylcellulose medium containing 3 U/mL recombinant human erythropoietin (Methocult M334, Stem Cell Technologies, Vancouver, BC, Canda) with 10% IMDM (Mediatech) and 0.2% FBS. In some cases, the cultures were supplemented with 15 ng/mL mouse BMP4 (R&D Systems). BFU-Es were counted after 8 days.

### Isolation of Murine Endothelial Cells

Kidneys from double-transgenic animals actively expressing myrAkt1 in the endothelium were minced and digested for 1–1.5 hours at 37°C with 2% Worthington type 1 collagenase in Dulbecco’s PBS supplemented with Ca^2+^/Mg^2+^. Digested tissues were filtered through a 100-micron cell strainer (BD Discovery Labware, Bedford, MA) and washed twice in 0.1% BSA in PBS, then incubated for 20 minutes at 4°C with 50 µl of magnetic beads (Dynal Biotech) that had been conjugated with anti-mouse CD31 antibody (BD Biosciences). Cells with beads attached were collected using an MPC magnet (Dynal Biotech, Grand Island, NY) and washed vigorously in 0.1% BSA/PBS, then plated in pre-coated Type I collagen (Cohesion, Palo Alto, CA) 100-mm tissue culture plates. Cells were grown in 4.5 mg/mL glucose DMEM with pyruvate and HEPES (Mediatech) supplemented with 25 mg Endothelial Cell Mitogen (Biomedical Technologies, Stoughton, MA), 100 ug/mL heparin, 20% fetal bovine serum, non-essential amino acids, and penicillin/streptomycin. After 2 days in culture, non-attached cells and excess beads were removed. Cell purity was assessed by 1,1-dioctadecyl-3,3,3,3-tetramethylindocarbocyanine perchlorate-acetylated low-density lipoprotein (DiI-acetylated LDL) (Biomedical Technologies) and CD31 staining. Protein levels were assessed by Western blot with antibodies against phospho-Akt1 (Ser473) (9271, Cell Signaling Technology, Danvers, MA), total Akt1 (9272, Cell Signaling Technology), and GAPDH (374, Millipore, Billerica, MA). Blots were incubated in 5% BSA at 1∶500 dilution for 3 hours at room temperature (phosphoAkt1, GAPDH) or overnight at 4°C (total Akt1), then incubated with secondary antibody (GE Healthcare, Waukesha, WI) at 1∶1000 for 1 hour at room temperature.

### Bone Marrow Transplants

Approximately 10^7^ unfractionated bone marrow cells from donor mice were transplanted into lethally irradiated recipients (9 Gy of radiation 24 hours previously) by tail vein injection. Mice were allowed to recover for 12 weeks, and were then removed from tetracycline for 1 week and analyzed.

### Splenectomies

Mice were anesthetized with an intraperitoneal injection of 0.1 mL of a mixture of ketamine (9 mg/mL) and xylazine (0.9 mg/mL) in physiological buffer per 10 grams mouse weight. Pain was relieved by subcutaneous meloxicam injection (5 mg/kg), prior to and 24 hours after splenectomy. The left back region was shaved and prepared with Betadine and ethanol. A 3–4 mm vertical incision was made in the back, parallel to the spine. Both splenic vessels were ligated with silk and the spleen was gently removed. The peritoneum was closed with dissolvable vicryl sutures and the skin was closed using wound clips. Wound clips were removed after 24 hours and the mice were allowed to recover for at least 21 days prior to removal of tetracycline.

### Statistical Analysis

The Student t test was used to analyze the significance of results. P<0.05 was considered significant.

## Results

### Transient B Cell Increase in Endothelial MyrAkt1-overexpressing Animals

**Figure 1:**
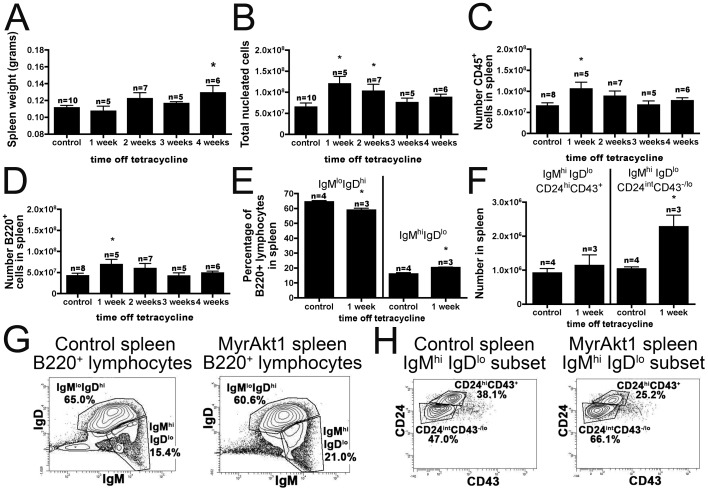
MyrAkt1 overexpression in endothelial cells in vivo leads to a transient increase in IgM^hi^IgD^lo^CD24^int^CD43^−/lo^ cells in the spleen. At 4 weeks off tetracycline, endothelial myrAkt1 mice displayed an increase in spleen weight (**A**) p<0.04. The total number of nucleated cells in the spleen increased transiently at 1–2 weeks but returned to normal at 3–4 weeks (**B**) p<0.03. The absolute numbers of CD45+ (**C**) and CD45+B220+ (**D**) cells displayed a transient increase at one week p<0.02. The relative percentage of IgM^lo^IgD^hi^ and IgM^hi^IgD^lo^ cells was altered at one week (**E**) p<0.02. Absolute numbers of B220^+^IgM^hi^IgD^lo^CD24^int^CD43^−/lo^ cells were increased at one week (**F**) p<0.01. Representative plots of B220^+^ fractionation by IgM and IgD (**G**), and B220^+^IgM^hi^IgD^lo^ fractionation by CD24 and CD43 (**H**).

To induce the expression of myrAkt1 in the endothelium of mice, tetracycline was removed from the drinking water of D4 double transgenic mice. As an additional control, double transgenic littermates were maintained on tetracycline and showed no differences from single transgenic littermate controls. Endothelial expression of myrAkt1 for 4 weeks resulted in a small but significant increase (15%) in spleen weight (**Fig. 1A**). The total number of nucleated cells showed a transient increase at one and two weeks (**Fig. 1B**). The total number of CD45+ leukocytes was also increased in the spleen at one week (**Fig. 1C**). No significant increases were observed in CD11b+, CD4+, or CD8+ subsets of CD45+ cells (data not shown), but the number of B220+CD45+ cells was significantly increased at one week (**Fig. 1D**). Characterization of B220+ cells with IgM and IgD showed a relative decrease of IgM^lo^IgD^hi^ (FO/B-2 cells) and a relative increase of IgM^hi^IgD^lo^ cells (**Fig. 1E,G**). Quantification showed no change in the absolute numbers of IgM^lo^IgD^hi^ cells (data not shown), indicating that the relative changes were due to an increase in the absolute number of cells in the IgM^hi^IgD^lo^ population. This population was further characterized with CD24 and CD43 staining, which showed an increase in the absolute numbers (**Fig. 1F**) and percentage (12.3±1.7 vs. 7.2±0.4 *p<0.002, **Fig. 1H**) of IgM^hi^IgD^lo^CD24^int^CD43^−/lo^ cells. This result is consistent with an increase in marginal zone B cells in the spleens of endothelial myrAkt1-overexpressing animals at one week off tetracycline.

### Splenic Erythropoiesis in Endothelial MyrAtk1-overexpressing Animals

The transient increase of B cells in the spleen at one week was insufficient to explain the increase in spleen weight over time. The increase in spleen weight at 4 weeks despite normalization of nucleated cell numbers (**Figs. 1A, B**) suggested an increase in non-nucleated red blood cells or fluid at later times and prompted further investigation of red cell differentiation in this model.

**Figure 2:**
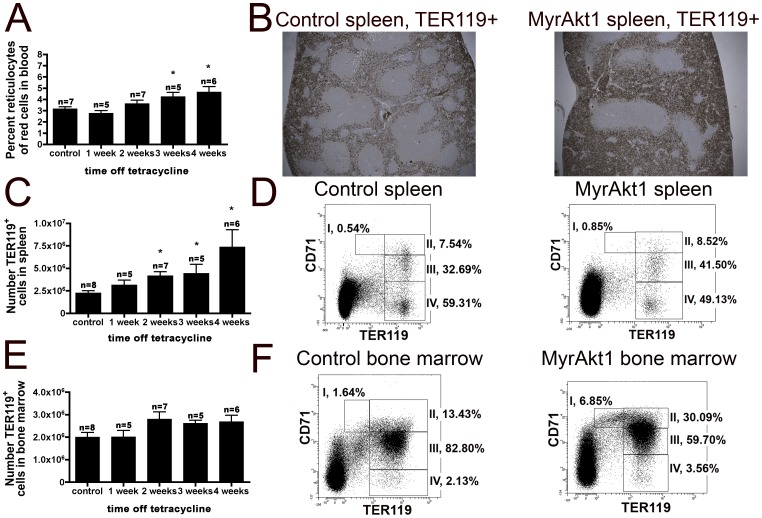
Increased spleen weight in myrAkt1 overexpressing animals is due to increased erythropoiesis. An increased percentage of reticulocytes was found in the blood at 3–4 weeks (**A**) p<0.02. Staining against TER119 revealed an increase in red cell area in the spleens of endothelial myrAkt1 compared with control mice (**B**) (5x). The total number of TER119^hi^ cells increased by 2 weeks and remained elevated in the spleen (**C**) p<0.04. Further characterization using CD71 and TER119 showed no differences in erythroid differentiation in the spleen (**D**). The total number of TER119^hi^ cells in the bone marrow remained constant (**E**), but myrAkt1 animals displayed an increase in stages I and II with a complementary decrease in III (**F**) p<0.007.

The percentage of circulating reticulocytes was increased at 3–4 weeks **(** **Fig. 2A** **).** Histological examination of the spleen revealed a disrupted architecture with increased red pulp containing TER119^+^ cells (a late erythroid marker expressed in erythroblast cells) (**Fig. 2B** **)**. By flow cytometry, we found that the number of TER119^hi^ nucleated red cell progenitors increased by 70% at two weeks in the spleen, and continued to increase over time (**Fig. 2C**). However, the absolute numbers of nucleated red cell progenitors remained small with respect to the total number of nucleated cells in the spleen and did not cause a significant change in total nucleated cell numbers (**Fig. 1B**). We further characterized these nucleated red cell progenitors using the method of Socolovsky et. al. [12], which broadly classifies erythropoietic progenitors by their relative expressions of CD71 (the transferrin receptor) and TER119 into four regions: TER119^med^CD71^hi^ (I, proerythroblasts); TER119^hi^CD71^hi^ (II, basophilic erythroblasts), TER119^hi^CD71^med^ (III, late basophilic and polychromatophilic erythroblasts), and TER119^hi^CD71^lo^ (IV, orthochromatophilic erythroblasts) (**Fig. 2D, F**). This analysis showed no difference in the relative percentages of the differentiating subsets in the spleen (**Fig. 2D**). The number of TER119^hi^ nucleated red cell progenitors in the bone marrow was constant (**Fig. 2E**), but myrAkt1 animals after one week off tetracycline displayed a transient but significant increase in the percentages of the earlier stages of I (5.76±1.30 vs. 2.62±1.55 *p<0.005) and II (31.18±3.00 vs. 14.92±6.09 *p<0.0005), compensated by a decrease in III (78.50±7.73 vs. 60.05±2.17 *p<0.007) (**Fig. 2F**). This pattern is consistent with other models of splenic erythropoiesis in which nucleated early red cell progenitors are produced in the bone marrow and migrate to the spleen for further expansion [5].

### Splenic Erythropoiesis is not Associated with Anemia or Hypoxia

**Figure 3:**
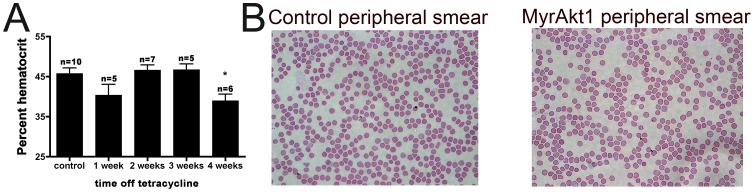
Splenic erythropoiesis is not triggered by anemia. Hematocrit remained unchanged until 4 weeks off tetracycline (**A**) p<0.01, and no hemolysis was observed in peripheral blood smears of endothelial myrAkt1 animals at 2 weeks off tetracycline as compared with controls (**B**) (100x).

Although evidence of splenic erythropoiesis was present in these animals at two weeks off tetracycline, the hematocrit remained unchanged until four weeks (**Fig. 3A**), when the mice began to develop other complications such as angiogenesis and edema [9], [10]. Blood smears showed no evidence of erythrocyte fragmentation (or other morphological abnormalities) at two or three weeks (**Fig. 3B**). Haptoglobin concentration and lactate dehydrogenase activity in serum were unchanged (data not shown). Oxygen saturation was normal at 3 weeks, and staining for hypoxia using Hypoxyprobe-1 revealed no visible increase in hypoxia in spleen, liver, or kidney at 3 weeks (data not shown). Together, these findings argue against a role for hemolysis or hypoxia in mediating the increased splenic erythropoiesis and reticulocytosis at early timepoints.

### Splenic Erythropoiesis is Independent of BMP4 and Erythropoieitin

**Figure 4:**
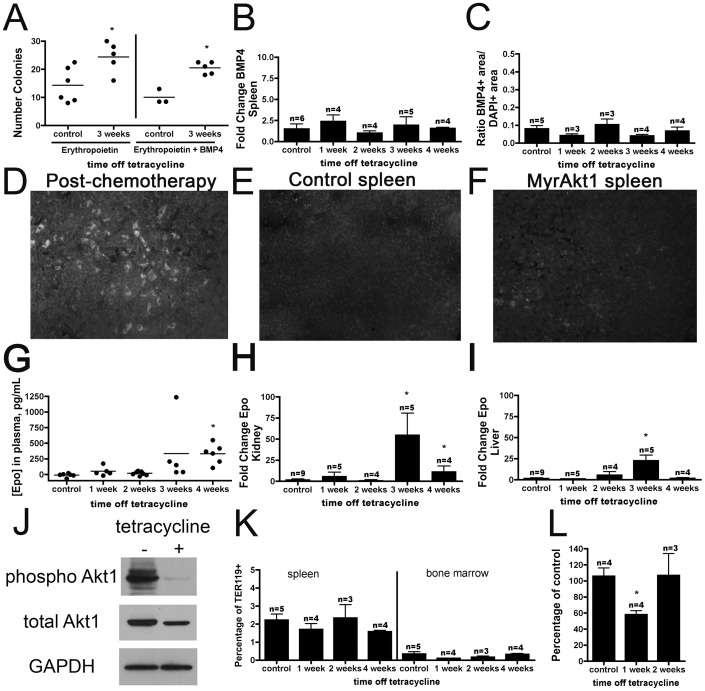
Initial splenic erythropoiesis is not mediated by erythropoietin or BMP4. Addition of BMP4 to methylcellulose cultures did not increase the number of colonies compared with erythropoietin alone (**A**). Levels of BMP4 mRNA (**B**) and protein (**C**) were unchanged in the spleen. Splenic BMP4+ macrophages recruited in response to chemotherapeutically-induced anemia (**D**) were not found in spleens of control (**E**) or myrAkt1 (**F**) animals (40x). Plasma erythropoietin protein was not significantly elevated until 4 weeks off tetracycline (**E**) p<0.0005, and levels of erythropoietin mRNA were not elevated until 3 weeks off tetracycline in the kidney (**F**) or liver (**G**) p<0.05. Endothelial cells isolated from myrAkt1 kidneys displayed downregulation of phosphorylated and total Akt1 in the presence of tetracycline (**J**). TER119^+^ erythroblasts showed no significant decrease in Annexin V positivity in spleen or bone marrow (**K**), but TUNEL staining showed a transient decrease in apoptotic nuclei in myrAkt1 spleens at one week p<0.006 (**L)**.

Other models of stress-induced splenic erythropoiesis have been shown to be dependent on BMP4 [3]–[6], [13]. To investigate the possibility that myrAkt1 overexpression in the endothelium resulted in increased differentiation of BMP4-dependent erythroid precursor cells in the spleen, we carried out in vitro colony forming assays. In the presence of erythropoietin, there was a 70% increase in BFU-E colonies from splenic cells plated from double transgenic animals off tetracycline for 3 weeks (**Fig. 4A**). Previous studies have shown that stress-induced erythropoiesis in the spleen is dependent on BMP4 [3]–[6], [13]. However, the addition of BMP4 to the methylcellulose assays did not result in a further increase in colony formation (**Fig. 4A**). Splenic levels of BMP4 mRNA (**Fig. 4B**) in endothelial myrAkt1 animals did not increase over time. Consistent with this result, immunohistochemistry against BMP4 in the spleen also failed to show any increase in overall levels of BMP4 protein (**Fig. 4C**) or numbers of BMP4-expressing macrophages that are present in spleens of anemic animals [14] (**Figs. 4D, E, F**) at any timepoint. Megakaryopoiesis was not affected, as evaluated by platelet counts in peripheral blood and numbers of CD41+ megakaryocytes in the spleen (data not shown).

Despite the early increase in the number of splenic TER119^+^ cells at 2 weeks in endothelial myrAkt1-expressing mice, plasma erythropoietin levels did not increase until 4 weeks off tetracycline (**Fig. 4G**). Tissue mRNA levels for erythropoietin were increased in the kidney (26-fold) and liver (13.9-fold) after three weeks off tetracycline (**Figs. 4H, I**). Erythropoietin levels in the kidney and liver followed a similar pattern to hematocrit (**Fig. 3A**), falling at 4 weeks (**Figs. 4H, I**) when the mice begin to develop other complications such as angiogenesis and edema [9], [10]. No increase in erythropoietin mRNA levels was observed in the spleen at any time point (data not shown).

To determine whether myrAkt1-expressing endothelial cells were producing erythropoietin, endothelial cells were isolated from the kidneys of double transgenic mice. Confluent plates were evaluated in the absence or presence of tetracycline. Tetracycline-mediated downregulation of total Akt1 and phospho-Akt1 was confirmed by Western blot (**Fig. 4J**). No erythropoietin protein was detected in the supernatants (data not shown).

One function of erythropoietin is to inhibit apoptosis of erythroid progenitors [15], but the TER119+ subset displayed no significant changes in Annexin V positivity in the spleen or bone marrow by flow cytometry (**Fig. 4K**). In another measure of apoptosis, TUNEL staining of the spleen did show a transient decrease in TUNEL+ apoptotic nuclei at one week off tetracycline (**Fig. 4L**). The transient nature of this decrease, combined with the lack of a concomitant decrease in Annexin V staining, suggests that apoptotic inhibition was not targeted to erythroblasts and is more likely related to the transient increase in splenic marginal zone B cells in this model.

### Splenic Erythropoiesis is Driven by Host Stroma, not Hematopoietic Progenitor Cells

**Figure 5:**
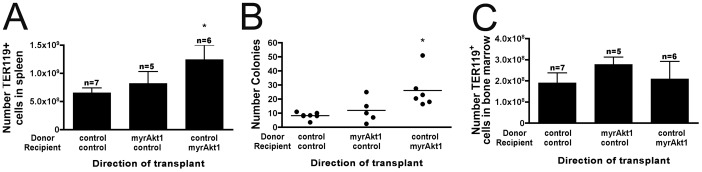
Splenic erythropoiesis in endothelial myrAkt1 mice is due to host and not stem cell genotype. Lethally irradiated control hosts rescued with endothelial myrAkt1 bone marrow did not display splenic erythropoiesis, in contrast with lethally irradiated endothelial myrAkt1 hosts rescued with control bone marrow, as measured by absolute number of TER119^hi^ cells in spleen (**A**) p<0.05 and number of BFU-Es from methylcellulose culture (**B**) p<0.005. Neither transplant resulted in an increase in TER119^hi^ cells in the bone marrow (**C**).

VE-cadherin has been shown to be expressed in certain hematopoietic progenitor cells [16]. To rule out a contribution of myrAkt1 from the hematopoietic stem cell compartment, we carried out bone marrow transplant experiments. Owing to the length of time of these studies, we chose the stronger-expressing D5 strain of endothelial myrAkt1 mice. This line displays a splenic phenotype by one week off tetracycline. Bone marrow transplants were performed from control to control, from endothelial myrAkt1 to control, and from control to endothelial myrAkt1 animals. Only the transplants into endothelial myrAkt1 hosts displayed an increased number of TER119^hi^ cells in the spleen by flow cytometry (**Fig. 5A**) and an increased number of BFU-Es in the spleen by methylcellulose culture (**Fig. 5B**). No changes were seen in the TER119^hi^ population in the bone marrow (**Fig. 5C**). Methylcellulose cultures co-seeded with cultured endothelial cells, enriched media from cultured endothelial cells, or media used to dissociate the spleens, did not produce BFU-Es, and no determination could be made about whether the effect of endothelial cells upon red cell progenitors is mediated by direct contact or secretion.

### Removal of Spleen Drives Increased Erythropoiesis to the Bone Marrow

**Figure 6:**
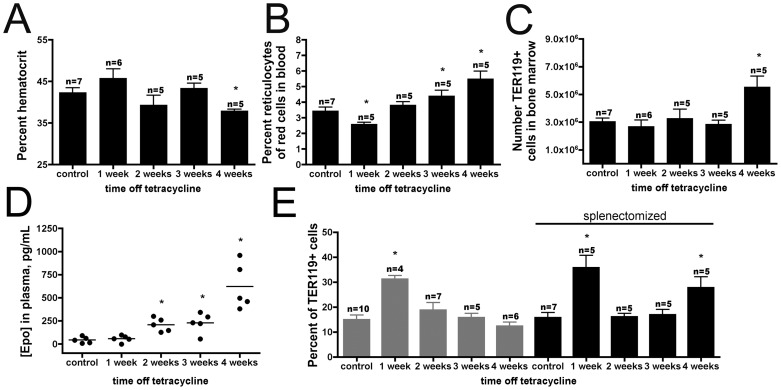
Removal of spleen caused a shift in non-steady state erythropoiesis to the bone marrow. Splenectomized endothelial myrAkt1 mice displayed the same trends in hematocrit (**A**) p<0.02, and reticulocytes (**B**) p<0.05, as non-splenectomized animals, but showed an increase in absolute number of TER119^hi^ cells in bone marrow (**C**) p<0.01, and an earlier increase in plasma erythropoietin protein (**D**) p<0.01. In contrast with myrAkt1 animals which display a transient increase in percentage of stage II erythroblasts followed by resolution (gray bars), splenectomized myrAkt1 animals displayed a second peak of stage II erythroblasts at 4 weeks (black bars (**E**)) p<0.03.

The onset of anemia (**Figs. 6A** **,** **3A**) and increased production of reticulocytes (**Figs. 6B** **,** **2A**) were unchanged in splenectomized endothelial myrAkt1 animals. However, the lack of a spleen appeared to drive the proliferative erythropoietic response to the bone marrow, with an increase of TER119^hi^ cells in the bone marrow at 4 weeks (**Fig. 6C**). Splenectomized animals displayed an earlier increase in plasma erythropoietin that may be related to the shift in proliferative erythropoietic tissue location (**Fig. 6D**). The lack of red cell recovery post-splenectomy was also associated with a second increase in the percentage of the differentiation stage II in the bone marrow at 4 weeks (**Fig. 6E**).

## Discussion

We have shown that over-expression of constitutively active Akt1 in the endothelium results in increased splenic erythropoiesis, which mimics a stress-like phenotype. The different effects on erythroblast populations in the spleen and bone marrow indicate specific roles for Akt1 in erythropoiesis in different tissues.

Previous studies have implicated a role for several extracellular and intracellular factors in mediating stress erythropoiesis. Among these are tissue hypoxia and erythropoietin. However, we were unable to detect any evidence of hypoxia in the myrAkt1-expressing mice. Moreover, the fact that the number of erythroid progenitor cells increased in myrAkt1 expressing spleens prior to any detectable change in the plasma erythropoietin levels argues against a role for enhanced erythropoietin signaling in our model. Other studies have demonstrated the importance of BMP4, stem cell factor (SCF) and hedgehog signaling in driving the expansion of stress erythroid progenitors in the spleen (reviewed in Paulson et. al. [17]). However, our findings that BMP4 levels are unchanged in the spleens of endothelial myrAkt1 mice and that the addition of BMP4 to the methylcellulose assays failed to further increase colony formation argue against a primary role for this factor in our phenotype. Adult ERK1^−/−^ mice develop an increased splenic erythropoiesis, which mimics a stress phenotype [18]. As with our study, this occurred in the absence of anemia. However, in contrast to our data, the stress erythropoiesis phenotype of ERK1^−/−^ mice was reproduced upon transplantation of ERK1^−/−^ bone marrow cells into wild-type recipients. Our work suggests that there may be multiple pathways that regulate splenic erythropoiesis under stress.

There is increasing evidence for a role of the hematopoietic microenvironment, or stroma, in mediating erythropoiesis. The stroma includes fibroblasts, osteoblasts, endothelial cells, adipocytes, and macrophages. Endothelial cells line the bone marrow and splenic vasculature. Bone marrow endothelial cells have been implicated in the maintenance of hematopoietic stem and progenitor cells as well as the differentiation of lineage-committed progenitors [11], [19], [20]. Interactions between endothelial cells and erythroid cells involve both endothelial-derived soluble signals as well as signals that depend on cell-cell contact [21]. In a recent study, knockdown of hypoxia inducible factor (HIF)-2α in mice was shown to result in normocytic anemia despite unaltered erythropoietin levels [22]. Transplantation analyses demonstrated that the defect affected the hematopoietic compartment in both spleen and bone marrow and lineage-specific rescue experiments localized the defect to the endothelium. HIF-2α was shown to induce the expression of vascular cell adhesion molecule (VCAM)-1, which in turn, was proposed to exert a paracrine effect on the differentiation of erythroblasts. Collectively, the data from that study suggest that endothelial HIF-2α plays an erythropoietin-independent, VCAM-1-dependent role in erythropoiesis. The findings of the current study also place the defect in the endothelial compartment, with different roles for bone marrow and splenic endothelium. However, myrAkt1-expressing mice did not express higher mRNA levels of VCAM-1 (data not shown).

Despite a significant increase in myrAkt1 expression both in the spleen and bone marrow [11], the effects on erythropoiesis were different in each tissue, with changes in differentiation in the bone marrow but changes in proliferation in the spleen. It is possible that the vascular niche differs between the spleen and bone marrow such that myrAkt1-expressing splenic endothelial cells secrete different trophic factors than bone marrow sinusoidal endothelial cells. A previous study showed that Akt1 activation in human endothelial cells derived from different sources results in similar patterns of gene expression [11]. However, the latter study did not examine endothelial cells from the spleen or assay for erythroid colony formation. Another possibility is that the different responses reflect differences in properties between differentiating erythroid cells in the spleen and bone marrow. For example, splenic cells have been shown to exhibit higher levels of Fas- and FasL-dependent apoptosis [23]. However, in our study, we did not observe any difference in the number of apoptotic splenic erythroid cells in myrAkt1 expressing mice. Further studies are required to determine whether or not other inherent differences in these populations of erythroid progenitor cells account for the spleen-specific response of endothelial myrAkt1.

Although the major hematopoietic effect in this model was on erythropoiesis, we did find a specific but transient increase in the number of putative marginal zone B cells in the spleen. The constant number of follicular B cells does not indicate a shift in relative commitment to marginal zone versus follicular B cells [24] but may be a reduction in apoptosis or increase in proliferation. The specificity of the effect may be due to the close proximity of the marginal zone to the red pulp and increased exposure to a secreted mediator; to the similarities between the fenestrated endothelium in the marginal zone and red pulp compared with the white pulp; or an increased sensitivity to the mediators among marginal zone B cells compared with follicular B cells. Marginal zone B cell development has been shown to be dependent upon non-hematopoietic cells in the spleen [25]. The temporary increase in marginal zone B cell numbers may be resolved after the initial event by the strong homeostatic mechanisms that control the size of B cell pools [26]–[28].
