# Correction: Overexpression of MyrAkt1 in Endothelial Cells Leads to Erythropoietin- and BMP4-Independent Splenic Erythropoiesis in Mice

**DOI:** 10.1371/annotation/207f3e91-451e-4508-afab-77f01dae3fe9

**Published:** 2013-06-18

**Authors:** Rebekah K. O’Donnell, Whitney E. Goldstein, Carole Perruzzi, Laura E. Benjamin, William C. Aird

The fifth author's name was spelled incorrectly. The correct name is: William C. Aird. The correct abbreviation in the Contributions section is: WCA.The correct citation is: O’Donnell RK, Goldstein WE, Perruzzi C, Benjamin LE, Aird WC (2013) Overexpression of MyrAkt1 in Endothelial Cells Leads to Erythropoietin- and BMP4-Independent Splenic Erythropoiesis in Mice. PLoS ONE 8(1): e55095. doi:10.1371/journal.pone.0055095. 

